# Supplementary material for: Improved MobileNetV2 crop disease identification model for intelligent agriculture
Source: PeerJ Comput Sci. 2023 Sep 25;9:e1595. doi: 10.7717/peerj-cs.1595 (PMC10557480; doi:10.7717/peerj-cs.1595)
Supplement: Supplemental Information 1 [file peerj-cs-09-1595-s001.docx]

Data Availability

Set the data source url：<https://data.mendeley.com/datasets/tywbtsjrjv/1>

The dataset derives from the plant_leaf_diseases_dataset_without_augout.zip in the web address. There are 39 different types of plant leaves and background images in the compression package, but our paper experiments use 25 different types of plant leaves. The specific usage is shown in Table 1.

**Table 1.** Data set

| type of data | Number | type of data | Number |
| --- | --- | --- | --- |
| Apple scab | 630 | Grape black rot | 1180 |
| Apple black rot | 621 | Grape esca | 1383 |
| Apple pine rust | 275 | Grape Leaf blight | 1076 |
| Apple health | 1645 | Grape health | 423 |
| Corn Cercospora leaf spot | 513 | Tomato Bacterial spot | 2127 |
| Corn Common rust | 1192 | Tomato early blight | 1000 |
| Corn northern leaf blight | 985 | Tomato late blight | 1909 |
| Corn health | 1162 | Tomato Leaf Mildew | 952 |
| Potato early blight | 1000 | Tomato Septoria leaf spot | 1771 |
| Potato late blight | 1000 | Tomato Spider mites Two spotted spider mite | 1676 |
| Potato health | 152 | Tomato Target Spot | 1404 |
| Tomato yellow leaf curl | 5357 | Tomato mosaic virus | 373 |
| Tomato health | 1591 |  |  |

The data set Information used to support the findings of this study are included within the article, and the PlantVillage data used to support the findings of this study are included within the supplementary information file.

The data used in this study is a publicly available dataset.
