# Supplementary figures and images for: Improved MobileNetV2 crop disease identification model for intelligent agriculture
Source: PeerJ Comput Sci. 2023 Sep 25;9:e1595. doi: 10.7717/peerj-cs.1595 (PMC10557480; doi:10.7717/peerj-cs.1595)

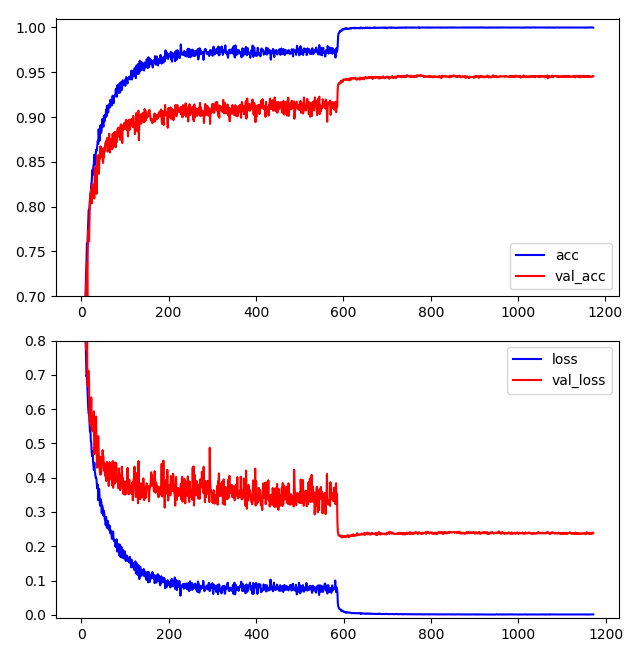

Supplement: Supplemental Information 2 [file peerj-cs-09-1595-s002.zip › bak/curve.png]

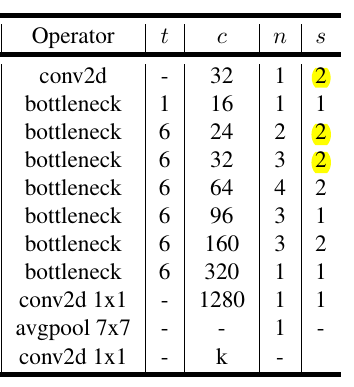

Supplement: Supplemental Information 2 [file peerj-cs-09-1595-s002.zip › bak/table.png]
